# Correction: Beyond ingredients: Supramolecular structure of lipid droplets in infant formula affects metabolic and brain function in mouse models

**DOI:** 10.1371/journal.pone.0321667

**Published:** 2025-04-01

**Authors:** Annemarie Oosting, Louise Harvey, Silvia Ringler, Gertjan van Dijk, Lidewij Schipper

In Fig 1, the description of panels B and D are incorrectly switched in both the figure and its caption. The description of panel B and D should have been “Ø 3-5μm” and “Ø 0.4μm” respectively. Please see the correct Fig 1 and its caption here:

**Fig 1:**
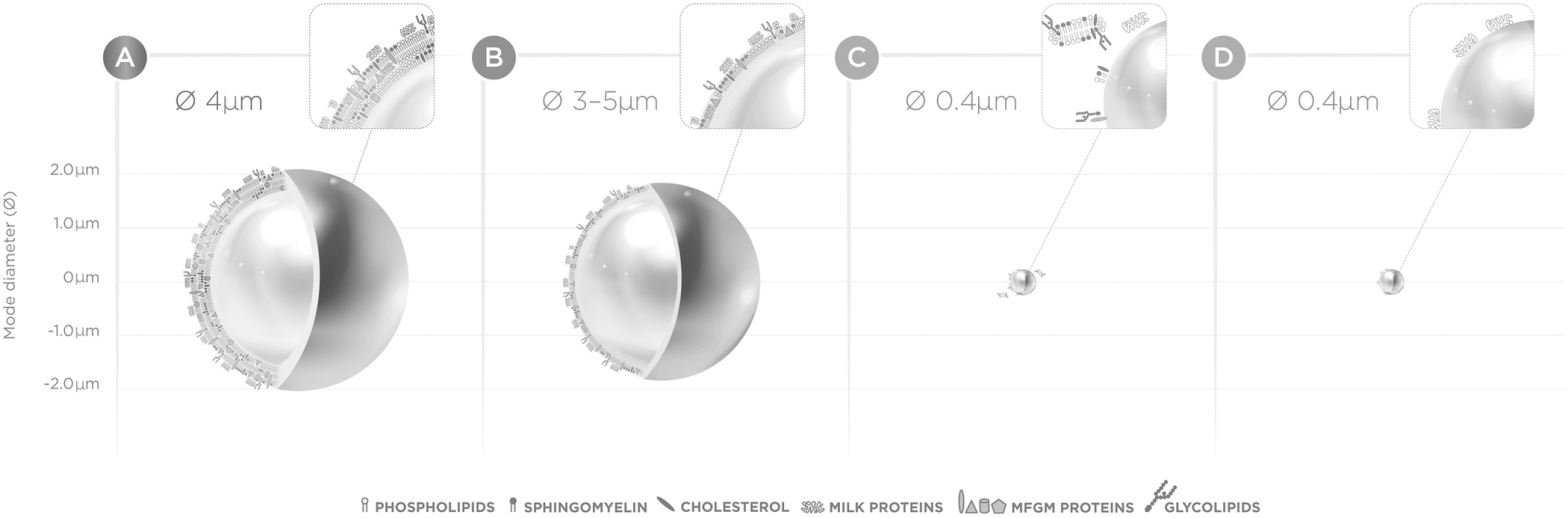
Schematic overview of lipid globules.

Schematic overview of lipid globules with tri-layer of MFGM in human milk (A), standard infant formula without MGFM but with milk proteins at the droplet interface (B), lipid droplet in standard infant formula with MGFM dry blended (C) and lipid droplet with MFGM fragments at the droplet interface (D; Nuturis). Visual representation of the size and ratios of different MFGM and milk protein molecules does not fully reflect reality.
